# Penitentiary Medicine Education in Italy: Medical Training and Scientific Research Perspectives

**DOI:** 10.3390/healthcare14213711

**Published:** 2026-11-02

**Authors:** Giulio Di Mizio

**Affiliations:** 1 Prison Medical Health Center, Intensive Assistance Service (SAI) Catanzaro Health Authority “U. Caridi” Correctional Center (Italian Ministry of Justice), 88100 Catanzaro, Italy; giulio.dimizio@unicz.it; 2 Forensic Medicine and Criminology, Law Department, University Campus “S. Venuta”, 88100 Catanzaro, Italy

**Keywords:** penitentiary medicine, correctional healthcare, medical education, health in prisons, medical ethics, healthcare workforce, public health policy

## Abstract

Penitentiary medicine in Italy suffers from limited academic and educational structuring. This is reflected in the absence of a uniform, nationally integrated university training pathway within medical curricula, as well as the fragmentation of scientific research in the field. The discipline, not being configured as an autonomous national academic and training track, relies on the integration of knowledge from various clinical, psychosocial, and legal disciplines, predominantly transmitted through on-the-job experiential learning. This status quo contributes to operational challenges, poor professional attractiveness, and high turnover rates among physicians in a context where specific training remains heterogeneous. This commentary analyzes the critical issues of the Italian system, identifies the main challenges of correctional healthcare, and proposes a theoretical framework outlining the fundamental principles of penitentiary medicine. It emphasizes the need for early, structured, and contextualized educational pathways covering ethical, legal, medico-legal, and penal execution aspects. Furthermore, an ideal educational backbone for correctional physicians is delineated, considering the daily clinical complexity characterizing this profession. A five-stage educational sequence (undergraduate to continuing education) and a roadmap assigning implementation responsibilities to universities, minis-tries, regions and scientific societies are also proposed.

## 1. Introduction

Penitentiary medicine is a highly complex healthcare sector characterized by unique clinical, psychological, and social needs. Incarcerated individuals represent a vulnerable population presenting a high prevalence of infectious diseases, psychiatric disorders, substance use disorders, and critical social conditions [1,2]. Healthcare management in correctional settings requires multidisciplinary skills alongside a robust understanding of the legal frameworks governing penal execution. Data at the European level consistently highlight the significant burden of mental health conditions, alongside critical issues regarding continuity of care and infectious disease management [3,4]. In Italy, however, penitentiary medicine is not formally recognized as an independent academic discipline with a dedicated national training curriculum. While this limited academic structure has not prevented localized educational initiatives, it has led to a fragmented offer that is inconsistently integrated into university programs, resulting in a learning process heavily reliant on the experiential transmission of operational practices. Only a few universities have established specialized master’s programs or advanced training courses, which remain insufficient to bridge the educational gap. Consequently, a uniform, nationally standardized, and systematically integrated university curriculum for medical students is currently lacking. The absence of a structured disciplinary and professional identity poses critical challenges to career attractiveness, advancement, ongoing professional development, and specialized competency acquisition. This scenario is further compounded by perceived occupational hazards, high attrition rates among practicing physicians, and heightened clinical risk. Concurrently, scientific research within this field remains fragmented and underdeveloped, underscoring the need for stable institutional support and interdisciplinary frameworks. The health burden of incarcerated populations is well known. Worldwide one in eight people in prison has major depression and around 4% have psychotic illness [5,6]; HIV prevalence, hepatitis C, hepatitis B and tuberculosis are consistently higher than in the general population [7]. Suicide rates in custody are high and after release excess mortality (especially first weeks post-release) [8] make prison health an integral part of public health [9,10]. This burden defines what correctional physicians need to have as competencies and is where any educational proposal should start from. In this paper “penitentiary medicine” refers to the medical discipline and its body of knowledge; prison healthcare refers to the organization and delivery of health services in custodial settings; prison and correctional healthcare are used interchangeably.

## 2. Methods and Analytical Framework

This commentary is based on narrative review of the literature combined with documentary analysis of institutional and regulatory sources. It is not a systematic review; its purpose is to provide critical synthesis supporting struc-tured educational proposal. Literature search. PubMed/MEDLINE, Scopus and Web of Science were searched for articles published between January 1990 and September 2026. Search strings com-bined terms related to the setting (“prison”, “correctional”, “carceral”, “peniten-tiary”, “detention”) with terms related to education and workforce (“medical edu-cation”, “curriculum”, “training”, “competencies”, “workforce”, “recruitment”, “burnout”), ethics (“dual loyalty”, “medical ethics”, “equivalence of care”) and governance (“governance”, “Ministry of Health”, “continuity of care”, “telemedi-cine”). Reference lists of retrieved articles were hand-searched for additional relevant studies. Inclusion criteria. Articles published in English or Italian were included if they dealt with at least one of the following: (i) education and training of physicians and other health professionals working in custody; (ii) workforce issues in prison healthcare (recruitment, retention, occupational stress); (iii) ethical and medico-legal aspects of clinical practice in prison; (iv) governance models of prison healthcare and their integration with public health; (v) health burden of incarcerated populations (as it defines the competencies required by correctional physicians). Studies on immigration detention or police custody were included only when their results were directly transferable to prison settings. Opinion pieces without substantive analytical contribution were excluded. Documentary analysis. Institutional documents issued by the World Health Organi-zation (WHO) Regional Office for Europe (including the Health in Prisons European Database (HIPED)) and Council of Europe, documents of the Italian Society of Penitentiary Medicine and Healthcare (SIMSPe), Italian legislative framework on prison healthcare and execution of sentences were examined to frame the Italian situation. Comparative approach. The comparison between Italy and other European countries (Section 4) is descriptive. It is based on the WHO/Europe survey of training opportunities for prison healthcare personnel [11], on mapping of prison healthcare governance across Council of Europe member states [12] and on published accounts of national experiences (particularly in the UK) [13,14,15]. No quantitative ranking of national systems was made.

## 3. Critical Issues and Discussion

### 3.1. Regulatory Intersection and Operational Ambiguity (DPCM 01/04/2008–Law 81/2014)

Following the reform process initiated by Legislative Decree 230/1999 that transferred prison healthcare management to the National Health Service (SSN) via the Prime Ministerial Decree (DPCM) of 1 April 2008 [16,17], through to Law 81/2014 [18]—which mandated the closure of Judicial Psychiatric Hospitals (OPGs)—healthcare workers have had to navigate an increasingly intricate regulatory landscape. The simultaneous presence of health, penal, and judicial regulations necessitates extensive inter-institutional coordination, often giving rise to interpretive and operational ambiguities in daily practice. This regulatory and organizational complexity creates an operational “gray zone” that exposes physicians, healthcare staff, correctional authorities, and the judiciary to administrative hurdles and liability risks. Consequently, the healthcare delivered within correctional facilities—specifically where clinical care intersects with judicial execution—holds strategic importance both for safeguarding prisoners’ health rights and for ensuring the lawful execution of sentences.

Since the Italian health system is organised on a regional basis, the 2008 reform put prison healthcare not in the hands of one national provider, but to the regional health systems and their local health authorities (ASL/ASP). Staffing models, contractual arrangements, availability of specialist care and organisation of mental health services in prison vary considerably across the country [19,20]. At the same time the Ministry of Justice still has responsibility for security, custody and prison environment while the Ministry of Health and Regions are responsible for care. This split jurisdiction which is common internationally [12,21] causes recurrent friction in Italy: escorts for external visits depend on custodial staff availability; clinical information flows between health and prison administrations are not standardised; operational protocols differ from one institution to another. The transfer of a detained patient to an external healthcare facility is one example of this complexity. Article 11 of the Penitentiary Act (Law 354/1975) [22] and Article 17 of Presidential Decree 230/2000 [23] state that such a transfer has to be authorized by the competent judicial authority, in urgent cases the prison director may order it and inform the authority afterwards. Let’s say we have a detained patient with chest pain at night; the physician must decide if the case is urgent enough for authorization, document that decision, notify prison director and escort with custodial staff while clinical time runs parallel to administrative time (a delay due to custodial logistics can have clinical impact and physician remains liable for both the clinical decision and timing thereof). Safe management of these situations requires knowledge of legal framework which is not taught in medical school yet.

### 3.2. Patient Perception and the “Hybrid” Role of the Correctional Physician

Despite the formal separation between healthcare functions and correctional administration, conducting medical activities inside a penal institution inevitably influences how inmates perceive the physician’s role. Patients may view the doctor as an extension of the correctional authority or fear that clinical disclosures could adversely affect their legal status, thereby compromising the therapeutic alliance, breeding distrust, or prompting malingering behaviors [1,24]. While maintaining clinical independence, the correctional physician exercises a public function that directly impacts the patient’s penal trajectory. Physicians certify fitness for custody or solitary confinement, contribute to assessments for sentence deferral on medical grounds, document vulnerabilities, and evaluate potential malingering aimed at avoiding judicial hearings or obtaining legal benefits. This dual responsibility highlights the need for deep legal knowledge, medico-legal competence, and a clear distinction between clinical care and correctional administration. Rather than creating a conflict of interest, this context demands that clinicians effectively operate at the intersection of healthcare, law, and sentence execution. Medical decisions carry direct and indirect implications for both clinical outcomes and legal proceedings. As such, penitentiary medicine requires advanced training, a solid ethical foundation, and specialized communication skills.

Dual loyalty conflicts cannot be eliminated but they can be reduced. The most effective structural measure is to assign different medical roles to different persons: the treating physician should not also do forensic assessments or certifications for custodial or disciplinary use [24]. Where separation of roles is not possible physicians should explain transparently to the patient what each assessment is for and the limits of confidentiality; document clinical decisions separately from information shared with prison administration; refer ethically complex cases to institutional ethics consultation. Specific training on dual loyalty has been feasible and useful for all categories of health staff [25] and recent reviews have provided practical mitigation strategies applicable both inside prisons and in community hospitals caring for patients in custody [26]. These strategies should be part of core curriculum.

### 3.3. Occupational Wellbeing of Prison Health Staff

Working in prison exposes health professionals to violence, threats and repeated contact with patients’ traumatic histories; high exposure to trauma has been documented among prison mental health staff and burnout and compassion fatigue have been associated with staff characteristics, trauma exposure and working environment [27]. Evidence specific to physicians is scarce but British experience shows that inadequate training and working beyond competence lead to poor recruitment and retention [14,15] so education also has a protective function: physicians who understand the legal framework and their institutional role are less exposed to uncertainty and moral distress. Training programmes should include stress management skills, recognition of se-condary traumatic stress and access to supervision and peer support.

### 3.4. Continuity of Care and Digital Health

Release from prison is a very risky period: mortality in the first two weeks after release was almost thirteen times higher than in the general population [8]. Continuity of care depends on the exchange of clinical information between prison and community services. Internationally incompatible health information systems in prisons and in the community have been identified as a major obstacle [21]. In Italy the prison clinical record is not always integrated with regional electronic health records, release planning is largely individual initiative. Digital health can address some of these barriers. Telemedicine in prisons is at least as effective as conventional care (good patient satisfaction) but success depends on local context [28]. Remote consultations reduce the need for escorted transfers to hospital (delays and security risks), extend specialist access to geographically isolated institutions [29]; exclusion of people in prison from monitoring of health coverage hides inequalities that integrated digital records could measure [30]. Physicians should therefore be trained in release planning, in shared clinical records use and telemedicine within the security constraints of custodial settings.

### 3.5. Critical Synthesis of Medical Education Needs

Educational preparation for physicians in correctional settings should begin early during undergraduate medical school and continue into early post-graduate clinical practice. Relying solely on post-employment, on-the-job training leaves technical, regulatory, ethical, and medico-legal skill acquisition to trial and error, increasing clinical risk, practitioner insecurity, and operational inefficiencies. Ethical, legal, and medico-legal dimensions form the foundation of correctional medical education. Without a thorough understanding of the legal standards governing healthcare for persons deprived of liberty, physicians cannot practice safely, responsibly, or with full awareness of their institutional role. Ultimately, daily clinical practice in prisons demands a comprehensive framework combining clinical care, public health, ethics, law, and risk management, going beyond the simple application of general clinical skills to a prison environment.

## 4. European Comparison: Educational Models and Governance

A comparative evaluation with other European countries reveals that while penitentiary medicine is rarely recognized as a standalone specialty, several nations have developed more structured educational frameworks than Italy. The European context here is the member states of the WHO European Region covered by the WHO/Europe survey of training opportunities for prison healthcare personnel [11] and Council of Europe member states mapped for prison healthcare governance (13 had transferred to Ministry of Health by October 2023) [12]. UK published experiences are used as reference for educational models [14,15]. The comparison is descriptive and not ranking national systems. According to a 2023 [11] WHO Regional Office for Europe survey, 31 postgraduate education and training programs dedicated to correctional healthcare staff were identified across the region. Core topics covered in these programs include human rights, healthcare delivery in custodial settings, continuity of care post-release, mental health, and addiction management. International training initiatives for prison healthcare workers are also available. These include WHO/Europe programs focused on infectious disease management, chronic disease care, risk management, and medico-legal ethics, often paired with short clinical observational placements in correctional facilities. Educational methods are typically interdisciplinary, combining theoretical coursework with practical exposure, though variations in course length and accessibility remain. In Italy, despite the 2008 transition of correctional healthcare to the SSN, educational offerings remain fragmented, non-systematic, and lack national standards, leaving training largely reliant on informal peer mentorship (Table 1).

## 5. A Proposed Educational Framework for Penitentiary Medicine

### 5.1. Rationale

The literature is consistent in showing that most physicians graduate without any exposure to care of incarcerated patients [15,31] and the UK had the same problem decades ago: prison doctors were difficult to recruit, poorly trained and often working beyond competence [13,14]. The British response (more prison placements for medical students and postgraduate trainees; development of an academic base for prison healthcare [14]) can serve as a model for Italy. Transferring prison healthcare to SSN in 2008 was necessary condition for clinical independence but institutional integration has not been followed by integration into medical education. As already observed in international debate change of governance does not remove constraints on clinical work in prison [32].

### 5.2. Competency Domains and Educational Sequence

A longitudinal pathway with five stages (Table 2) is proposed (more specialized objectives towards the end): human rights, equivalence of care and medical ethics (including dual loyalty management); legal and medico-legal framework of penal execution; epidemiology and clinical management of conditions prevalent in prison (mental disorders, substance use disorders, infectious and chronic diseases); emergency care and clinical risk management in a custodial setting; continuity of care, transitional care and digital health; professional resilience and occupational wellbeing.

Stages 1 and 2 do not need new courses or credits: they can be built into existing teaching in Legal Medicine and Public Health (which already cover the relevant legal, ethical and epidemiological principles). Stage 5 is particularly important for physicians already in service and can be delivered within the national continuing medical education system (Educa-zione Continua in Medicina ECM). Structured setting specific training has been shown to be possible elsewhere as well (dual loyalty training given to all health staff in New York City jail system [25]).

### 5.3. Training the Trainers

The main problem is that faculty with direct experience of prison healthcare are rare. Three solutions are proposed: (i) involvement of experienced prison physicians as adjunct lecturers and clinical tutors; (ii) train-the-trainer programmes developed by universities together with scientific societies (in particular SIMSPe); (iii) sharing teaching materials, case libraries and assessment tools between universities so that medical schools without local expertise can adopt a common core.

### 5.4. Barriers to Implementation

Several obstacles must be acknowledged: medical curricula are already saturated and since there is no scientific-disciplinary sector dedicated to penitentiary medicine universities do not have an institutional “owner” of penitentiary medicine (faculty expertise is limited and unevenly distributed). Clinical placements need formal agreements between universities, health authorities and Prison Administration as well as security procedures for students’ access to prisons; regional heterogeneity in prison healthcare organisation makes it difficult to guarantee comparable training placements across the country. For these reasons the proposal is incremental: it builds on existing courses and structures rather than on new specialties or credits.

## 6. Conclusions and Policy Recommendations

Penitentiary medicine requires clearer academic recognition, dedicated university training options, and sustained investment in scientific research. The immediate goal need not be the creation of an independent medical specialty, but rather the establishment of a core competency curriculum integrated into both undergraduate medical education and postgraduate training. Implementation requires, coordinated action by several actors, as summarized in Table 3.

Establishing a standardized, interdisciplinary educational framework is essential to adequately prepare future correctional physicians, make the profession more attractive, improve healthcare quality for incarcerated populations, and align clinical care with institutional safety within the Italian prison system.

## Figures and Tables

**Table 1:** Educational and organizational features of penitentiary medicine: Italy and the European context.

Dimension	Italy	European Context
Governance of prison healthcare	Transferred to the SSN in 2008; delivered by regional health systems	Transferred to the Ministry of Health in 13 Council of Europe member states; in others, responsibility remains with the Ministry of Justice or is shared [12]
Integration into medical education	No mandatory content in the medical degree; institutional integration into the SSN has not been followed by educational integration	Heterogeneous; optional content within public health, forensic medicine, or community medicine; placements for students and trainees promoted in the UK [14]
Postgraduate training	Few master’s programs and advanced courses, not coordinated at the national level	31 postgraduate programs identified across the WHO European Region, varying in duration, methods, and access [11]
Clinical placements	Not included in national curricula; local initiatives only	Observational placements in some countries
Teaching methods	Mainly experiential, on the job	Case-based, interdisciplinary, e-learning, and blended formats
Core topics	Mental health, addiction, infectious diseases, emergencies, legal and medico-legal aspects	Human rights, delivering care in prison, mental health, addiction, infectious and chronic diseases, continuity of care [11]
National standards and monitoring	No minimum national curriculum	International frameworks available; prison health systems monitored through HIPED, which collects data on prison health systems and outcomes rather than on training [2]

Notes: Source: Author’s elaboration based on WHO Regional Office for Europe documentation, the literature cited, and Italian regulatory frameworks. HIPED: Health in Prisons European Database, available online: https://www.who.int/data/region/europe/health-in-prisons-european-database-(hiped) (accessed on 23 October 2026).

**Table 2:** Proposed sequence of penitentiary medicine education in Italy.

Stage	Setting	Content	Format
1. Undergraduate, pre-clinical years	Within existing courses of Legal Medicine and Public Health/Hygiene	Domains 1–2: right to health, equivalence of care, dual loyalty, legal status of the detained patient, epidemiology of incarcerated populations	Lectures and case-based seminars; no new course required
2. Undergraduate, clinical years (5th–6th year)	Elective clinical rotation in prison health services	Domains 3–5: clinical care in prison, communication, confidentiality, emergency pathways, release planning	Supervised observational placement (1–2 weeks); case discussion with prison physicians
3. Postgraduate training	Rotations within residency programs (Legal Medicine, Psychiatry, Infectious Diseases, Public Health, Emergency Medicine) and the General Practice training course	Domains 2–5 at specialist level: fitness for detention, medico-legal certification, management of complex patients	Structured rotation in prison health services, with a tutor
4. Advanced training	Second-level university master’s programs and advanced courses	All domains, including healthcare management and inter-institutional coordination	Interdisciplinary curriculum (medicine, law, psychology)
5. Continuing education	Local health authorities (ASL/ASP) and scientific societies	Mandatory induction for newly hired physicians; periodic updates; dual loyalty training; staff wellbeing	Induction program with mentoring; blended e-learning; simulation

**Table 3:** Proposed roadmap and allocation of responsibilities.

Actor	Proposed Action
Ministry of University and Research and medical schools	Include core penitentiary medicine objectives in the national learning outcomes of the medical degree; promote elective clinical rotations in prison health services
Ministry of Health	Recognize penitentiary medicine as a priority area for continuing medical education (ECM); support national training standards
Ministry of Justice (Department of Penitentiary Administration)	Facilitate students’ and residents’ access to prisons through framework agreements with universities and health authorities
Regions and local health authorities (ASL/ASP), through the State–Regions Conference	Guarantee mandatory induction and continuing education for prison health staff; reduce regional disparities in training opportunities
Scientific societies (SIMSPe)	Develop the core curriculum, train-the-trainer programs, and shared teaching materials in partnership with universities

## Data Availability

No new data were created or analyzed in this study. Data sharing is not applicable to this article.
